# eHUGS: Enhanced Hierarchical Unbiased Graph Shrinkage for Efficient Groupwise Registration

**DOI:** 10.1371/journal.pone.0146870

**Published:** 2016-01-22

**Authors:** Guorong Wu, Xuewei Peng, Shihui Ying, Qian Wang, Pew-Thian Yap, Dan Shen, Dinggang Shen

**Affiliations:** 1 Department of Radiology and BRIC, University of North Carolina at Chapel Hill, Chapel Hill, NC, 27599, United States of America; 2 Department of Electrical & Computer Engineering, Texas A&M University, College Station, TX, 77843, United States of America; 3 Department of Mathematics, School of Science, Shanghai University, Shanghai, 200444, China; 4 Med-X Research Institute, Shanghai Jiao Tong University, Shanghai, 200240, China; 5 Department of Mathematics and Statistics, University of South Florida, Tampa, FL, 33620, United States of America; 6 Department of Brain and Cognitive Engineering, Korea University, Seoul, Republic of Korea; Beijing University of Technology, CHINA

## Abstract

Effective and efficient spatial normalization of a large population of brain images is critical for many clinical and research studies, but it is technically very challenging. A commonly used approach is to choose a certain image as the template and then align all other images in the population to this template by applying pairwise registration. To avoid the potential bias induced by the inappropriate template selection, groupwise registration methods have been proposed to simultaneously register all images to a latent common space. However, current groupwise registration methods do not make full use of image distribution information for more accurate registration. In this paper, we present a novel groupwise registration method that harnesses the image distribution information by capturing the image distribution manifold using a hierarchical graph with its nodes representing the individual images. More specifically, a low-level graph describes the image distribution in each subgroup, and a high-level graph encodes the relationship between representative images of subgroups. Given the graph representation, we can register all images to the common space by dynamically shrinking the graph on the image manifold. The topology of the entire image distribution is always maintained during graph shrinkage. Evaluations on two datasets, one for 80 elderly individuals and one for 285 infants, indicate that our method can yield promising results.

## Introduction

Since the advent of Magnetic Resonance Imaging (MRI), many imaging-based studies have been initiated to study structural variations within a population, between populations, and across different times of the same population [1–5]. In these studies, image registration is key for removing structural variations that confound the analysis of group differences or changes, e.g., those associated with brain disorders [6–8]. In particular, subtle changes may be elusive in cases where image registration is not performed with sufficient accuracy.

Many pairwise registration methods have been developed for registering a group of images to a template image [9–14]. However, template selection is not a trivial task [15, 16] and, if done improperly, will bias the subsequent statistical analysis. To deal with this issue, groupwise registration methods have been recently proposed to align all images jointly onto a common space, without the need of explicitly specifying the template. The objective function in groupwise registration aims to minimize *either* the overall intensity difference [17] *or* the entropy of joint intensity distributions across all images [18, 19]. To optimize the large-scale objective function in groupwise registration, efficient gradient-based Gauss-Newton optimization is proposed in [20]. A hierarchical groupwise registration mechanism is also used in [21] by selecting the key points in the image and only letting the key points drive the entire groupwise registration. The advantage of groupwise registration over pairwise registration has been widely demonstrated in the literature [15, 17, 19, 20, 22–24].

Many existing groupwise registration methods require an explicitly defined target of registration. For example, Joshi et al. [25] proposed an efficient groupwise registration algorithm by alternating between two steps: (1) register all images, separately, to a tentatively estimated group-mean image, and (2) update the group-mean image by averaging all registered images. This groupwise registration method works well with the datasets involving only small structural variations. However, for the datasets with large and complex variations, this method could result in a fuzzy group-mean image and thus decrease the registration accuracy. To solve this problem, Wu et al. (2011) proposed a sharp-mean based groupwise registration method by using a patch-based weighted-averaging method. However, a common limitation of these methods is that registration needs to be performed with respect to the group-mean image, regardless of whether the appearance of an individual image under registration is significantly different from the group-mean image or not. These methods are hence limited in dealing with the datasets with large and complex structural variations.

On the other hand, the image distribution information has been shown useful in guiding groupwise registration. For example, Wang et al. (2010) has demonstrated that the improved registration accuracy can be obtained by clustering the images in a population into several subgroups and then registering each of the subgroups with groupwise registration. Since images in a subgroup are similar in appearance, accurate registration can be obtained with relative ease. After within-subgroup registration, a representative image from each subgroup can be used for between-subgroup registration. However, one limitation of this method is that the within-subgroup distribution information is not specifically used for groupwise registration.

We developed an approach, called ABSORB (Atlas Building by Self-Organized Registration and Bundling) [17], to leverage image distribution information to guide groupwise registration. In ABSORB, each image is registered to a small number (e.g., *m* = 3∼5) of neighboring images, which are similar in appearance. Each image is then spatially transformed using the average of the *m* resulted deformations. This procedure is applied to all images iteratively. In this way, ABSORB avoids the need to register two images with large structural differences. However, the value of *m* is empirically determined in ABSORB and then fixed throughout the whole registration process. In addition, ABSORB is not aware of the image distribution of the entire dataset, but only a few neighbors.

To combine the advantages of above-mentioned hierarchical registration framework and ABSORB, we further proposed an approach called HUGS (Hierarchical Unbiased Graph Shrinkage) for groupwise registration [23]. In HUGS, we characterize the image distribution of the entire dataset by using a graph with its nodes representing the images and its edges representing the degree of similarity between the images. Only similar images are connected on the graph. The registration of all images to a hidden common space is formulated as a dynamic graph shrinkage problem, where all nodes move closer to each other along the edges. Since both global and local image distribution information is encoded in the graph, the topology of the entire image manifold can be preserved more accurately throughout registration.

Despite being more accurate, HUGS is still limited in dealing with heterogeneous datasets since a single simple graph is often insufficient in modeling complex image distributions. HUGS uses a simple threshold-based method to construct the graph by connecting image pairs with distances smaller than the given threshold. Given a heterogeneous dataset, the threshold has to be significantly relaxed in order to ensure that all images are connected on the graph, which often results in an over-connected and less-efficient graph. This will also cause many unnecessary registrations between dissimilar image pairs, which not only significantly increases the computation time but also results in considerable registration errors.

In this paper, we present a novel groupwise registration method that inherits the advantages of all our previously developed methods but, at the same time, addresses their limitations. Specifically, we introduce an improved version of HUGS, called enhanced HUGS or eHUGS, to deal with the registration of heterogeneous datasets by constructing a hierarchical graph to model the complex image distribution. The main idea is that we first represent the manifold of the population of brain images by a graph that characterizes the affinity or similarity between any two images. If two images are structurally similar, they are connected by an edge on the graph. This graph is used to help avoid the need to register two images with large difference. Since on the graph the images are represented as nodes and the distances between images are represented as edges, the groupwise registration problem then becomes a graph shrinkage problem, where the goal is to shrink the edges of the graph by progressively registering images adjacent on the graph. To construct a hierarchical graph, the images are first clustered into several subgroups via affinity propagation [26], which can group images into subgroups based on a set of automatically determined representative images. A representative image is identified to represent each subgroup, and the distribution in each subgroup is modeled using a graph. Then, the distribution of all these “low-level” graphs is captured by using “high-level” graphs that model the distribution of the representative images from all subgroups. After building the hierarchal graph, dynamic graph shrinkage is finally employed to register all images to the common space.

In the experiments, we first tuned the parameters and validated the registration performance of eHUGS by using 20 longitudinal series of images [27], each with 4 time points. After that, we applied eHUGS to 285 images of infants with ages ranging from 2 weeks to 2 years. We evaluated the quality of infant atlases generated by averaging those registered images in the common space. The results indicate that eHUGS yields greater registration accuracy than other groupwise registration methods, reflected by its built infant atlases with much clearer anatomical details.

### Ethics Statement

This study does not involve human subjects or samples. The individuals in this manuscript have given written informed consent at the time of enrollment for imaging and completed questionnaires approved by each participating sites. The authors have obtained approval from the ADNI Data Sharing and Publications Committee to use the data. The authors confirm that the data was analyzed anonymously.

## Materials and Methods

Given a large dataset of heterogeneous brain MR images, our goal is to simultaneously register all images to a hidden common space. The group-mean image does not need to be explicitly specified, thus avoiding concerns of how the low quality of group-mean images might undermine registration accuracy [22]. At the same time, the topological distribution of the entire image dataset will also be preserved throughout registration.

To achieve the above goal, we propose a hierarchical graph to model the distribution of all images in the dataset, and then attain groupwise registration by shrinking the graph dynamically until all images are warped to the hidden common space. For clarity and conciseness, we use ***I*** = {*I*_*i*_|*i* = 1, …, *N*} to denote a group of *N* images that are distributed on the manifold. The term *d*_*i*,*j*_ denotes the distance between image *I*_*i*_ and image *I*_*j*_. Since our focus is on deformable image registration, all images in the dataset are first affine-registered using FLIRT [28].

### Overview of eHUGS

**Fig 1:**
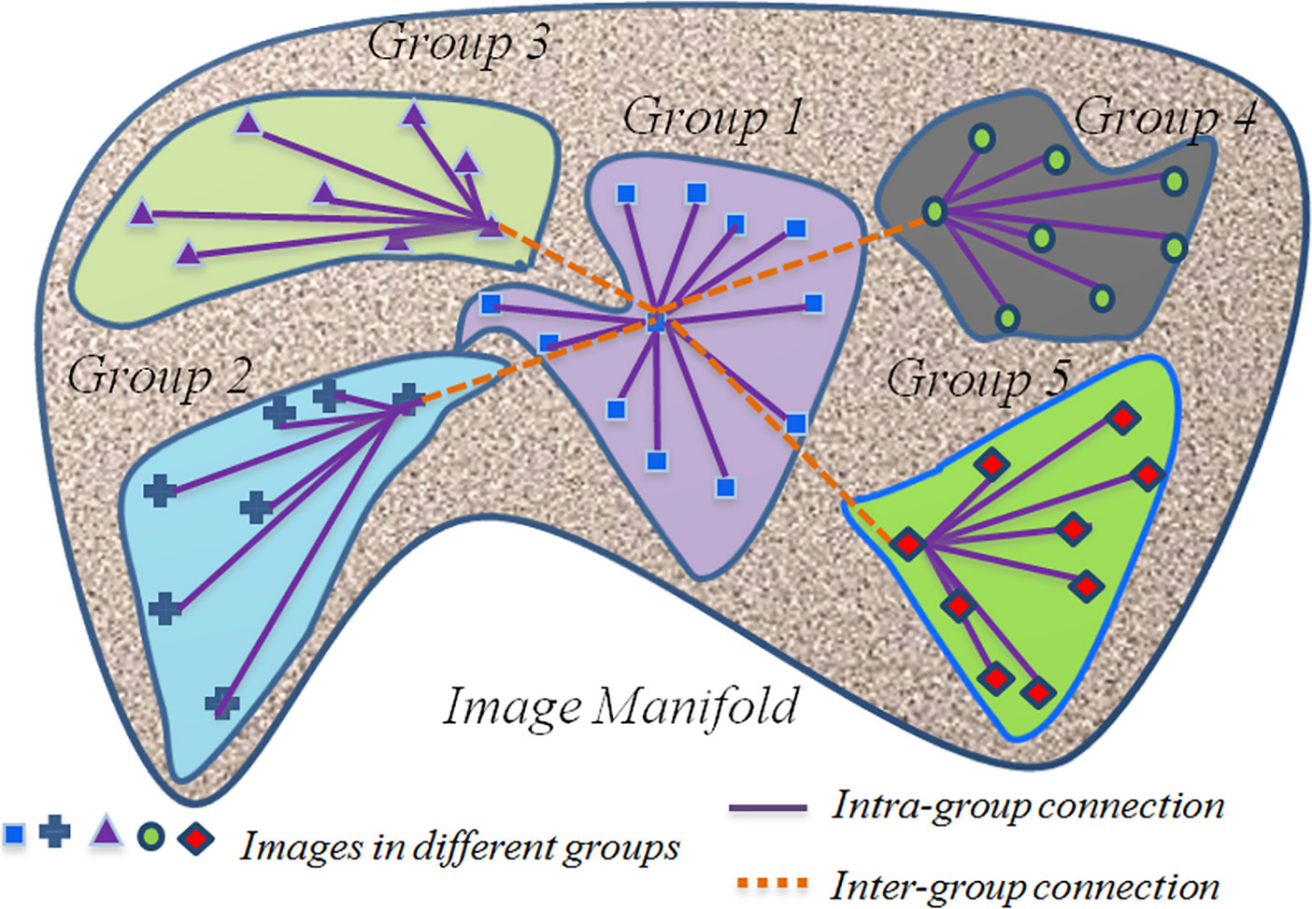
An illustration of Enhanced Hierarchical Unbiased Graph Shrinkage (eHUGS) method. In this example, the images are clustered into 5 subgroups based on their similarity. Then a hierarchical graph is constructed with each low-level graph (indicated by solid lines) describing the image distribution in each subgroup, and each high-level graph (indicated by dashed lines) encodes the interaction between the subgroups. Groupwise registration is formulated as the dynamic shrinkage of this hierarchical graph.

An overview of eHUGS is provided in Fig 1. eHUGS is an iterative method to deform all images together gradually as the graph shrinks. First, image similarity is computed based on a current set of deformed (or initially the affine transformed) images. Then, a hierarchical graph representing the distribution of the deformed images is built based on manifold *M*, which is determined using image clustering. In contrast to HUGS, eHUGS partitions the heterogeneous image group into several subgroups. Here, each subgroup contains only the homogeneous data with images of similar appearance. A low-level graph is then constructed to capture the distribution of images in each subgroup. These low-level graphs are further connected using the high-level graphs. Each image is allowed to deform only towards its connected counterparts in this hierarchical graph, progressively bringing the images closer to each other.

Groupwise registration is carried out by shrinking the graph gradually to deform all images to the hidden common space. Graph shrinkage is accomplished by dynamically and synchronously warping each image towards its connected neighbors in the graph. To cater to the hierarchical structure of the graph, we apply graph shrinkage sequentially for different levels. It is worth noting that more levels may be used to better represent the image distribution if needed, but will increase the computation time significantly. For our datasets, we found that using two levels is sufficient for reasonable representation of data, as well as for reasonable registration accuracy. More specifically, in the low level, each subgroup shrinks as all member images agglomerate. In the high level, the representative images of the subgroups are used to drive each subgroup closer to the common space. Graph shrinkage is executed alternatively throughout different levels until all images are registered to the common space. In what follows, details for both constructing the hierarchical graph and executing graph shrinking are provided.

### Construction of Hierarchical Graph

Graph construction is key to the success of eHUGS. As shown by Ying et al. [23], the graph needs to satisfy three criteria: (1) all images must be connected by the graph to guarantee that all images can be warped to a common space; (2) the number of edges in the graph needs to be minimized to reduce the computation load and also make graph shrinkage efficient; (3) the graph needs to capture the image distribution sufficiently well to ensure good registration accuracy.

We employ the hierarchical graph to capture the distribution of heterogeneous image data more accurately. Our graph reflects the relatively small variation within each subgroup as well as the large variation between subgroups. This is inspired by the work of Wang et al. [29], where the entire dataset of images are clustered into several subgroups based on the similarity between images. We follow a similar approach and derive the hierarchical graph based on the outcome of image clustering. First, the whole dataset of images are separated into several subgroups, each comprising similar images. Second, a representative image is selected from each subgroup to produce a high-level graph.

**Fig 2:**
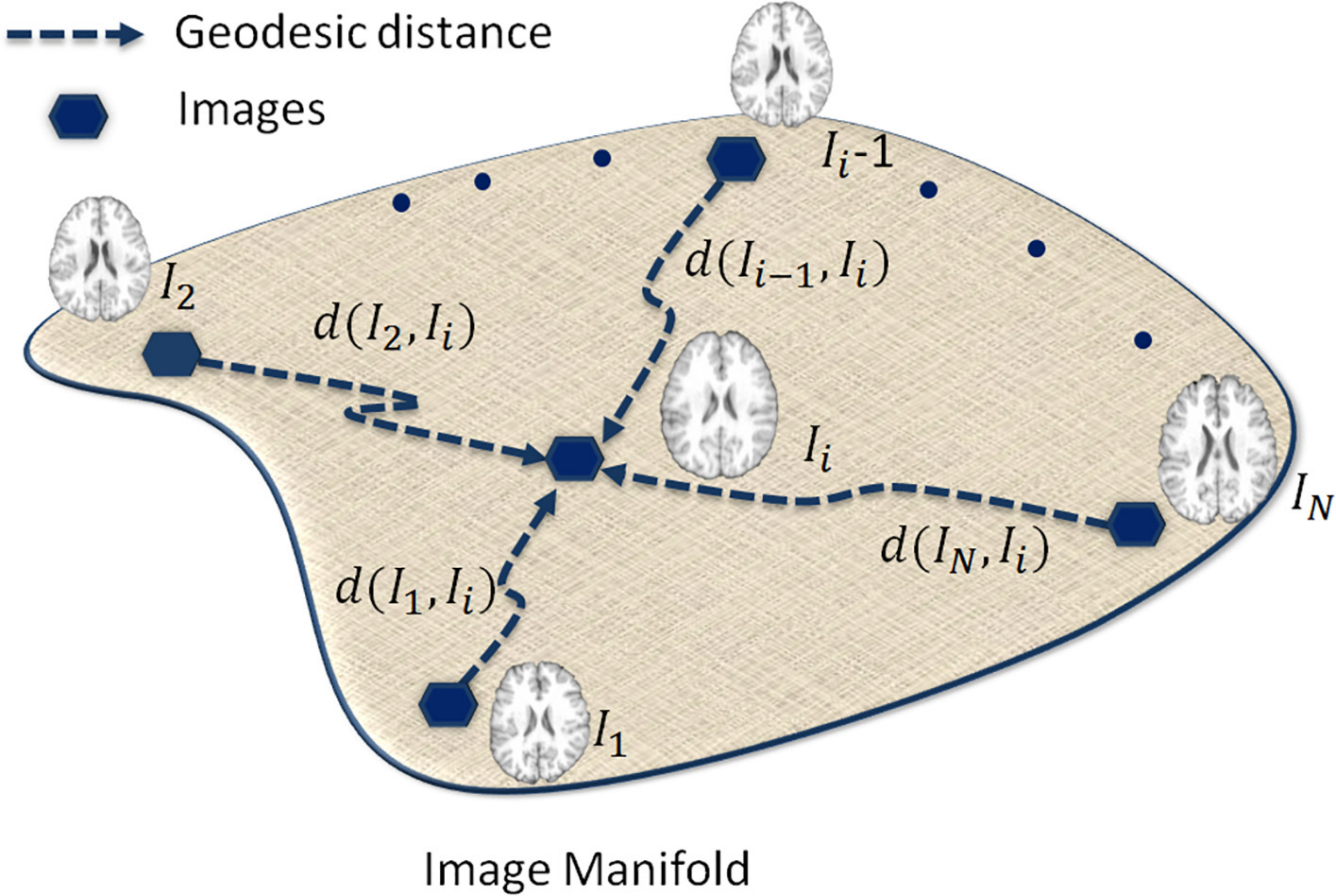
Illustration of images residing on a high dimensional manifold and connected via the geodesic paths. The geodesic distances between image *I*_*i*_ and other images are shown by dash arrows.

As illustrated in Fig 2, a set of *N* images ***I*** = {*I*_*i*_|*i* = 1, …, *N*} reside in a manifold *M*. The distance *d*_*i*,*j*_ for each pair of images *I*_*i*_ and *I*_*j*_ is defined as the sum of squared differences (SSD) *d*_*i*,*j*_ = ‖*I*_*i*_ − *I*_*j*_‖^2^. Note that there are many other choices for defining image similarity/distance, such as mutual information (MI) [30] and normalized mutual information (NMI) [31]. Our choice of SSD is simply for the sake of computational efficiency. Images with greater similarity tend to be spatially close to each other on the manifold. Since it is easier to register images with similar anatomical structures, the graph is constructed by arranging similar images into the common subgroups. The construction of the graph is accomplished by data-driven clustering as detailed next.

#### Data-Driven Image Clustering

A data-driven approach is used to cluster images into different subgroups based on their distances on the manifold. Image pairs with small distances tend to be clustered into the same subgroup, while images with large distances tend to be clustered into different subgroups. Methods such as *k*-means clustering [32] and affinity propagation [26] can be used. We opt for affinity propagation because, unlike k-means clustering, it does not require the explicit specification of the number of clusters.

An affinity matrix of similarities between all pairs of images is required by affinity propagation. We first apply affine registration to align all images to a common space, and then populate the *N* × *N* affinity matrix *S* whose elements *s*_*i*,*j*_ are defined as the negative SSD, i.e., *s*_*i*,*j*_ = −*d*_*i*,*j*_ = −‖*I*_*i*_ − *I*_*j*_‖^2^. We also set the preference value *p* in affinity propagation as below, which controls the likelihood of an image as a subgroup exemplar (representative image) and also eventually influences the number of exemplars:

$$p=\frac{1}{N^{2}}\sum_{j=1}^{N}\sum_{i=1}^{N}s_{i,j}=\frac{1}{N^{2}}\sum_{j=1}^{N}\sum_{i=1}^{N}{-d}_{i,j}=\frac{1}{N^{2}}\sum_{j=1}^{N}\sum_{i=1}^{N}-{\left\|I_{i}-I_{j}\right\|}^{2}.$$ (1)

In general, small preference value results in the small number of representative images. Since we do not have any priori knowledge on the observed images, we use the same value of *p* for all images, so that all images have equal possibility of becoming the representative image in each subgroup.

We further construct a *N* × *N* symmetric connection matrix *E* to encode the graph. If image *I*_*i*_ and image *I*_*j*_ is connected, we set the connection matrix *E*’s element *e*_*i*,*j*_ = 1; otherwise, *e*_*i*,*j*_ = 0. All diagonal entries in *E* are set to 0 so that no image is self-connected.

Specifically, we apply the following steps to build the graph:

1. Cluster the *N* images ***I*** = {*I*_*i*_|*i* = 1, …, *N*} into *Ω* subgroups *G*_*α*_ (*α* ∈ {1, …, *Ω*} and ${⋃}_{\alpha =1}^{\Omega }G_{\alpha }=I$) by using affinity propagation.
2. Determine the global center image *I*_*o*_ of the dataset,
  $$I_{o}=\underset{I\in I}{arg\;min\;}\sum_{i=1}^{N}{\left\|I_{i}-I\right\|}^{2},$$ (2)
  to approximate the hidden common space.
3. Within each subgroup *G*_*α*_, a representative image $I_{i_{\alpha }}$ is determined as the one nearest to the global center image *I*_*o*_. Thus, with *Ω* subgroups, we have *Ω* representative images: $\left\{I_{i_{\alpha }}|\alpha =1,\ldots ,\Omega \right\}$. Particularly, when the subgroup includes the global center image *I*_*o*_, its representative image which is the nearest to the global center image *I*_*o*_ is the global center image *I*_*o*_ itself. Thus, we have $I_{o}\in \{I_{i_{\alpha }}|\alpha =1,\ldots ,\Omega \}$. The advantage of choosing the image nearest to the global center for delegating the whole subgroup is that this (representative) image can make all other images in the whole subgroup deform easily towards the global center.
4. For each subgroup *G*_*α*_, intra-group connections are made between image $I_{i_{\alpha }}$ and all other member images. For any pair of images *I*_*i*_ and *I*_*j*_ ∈ *G*_*α*_ with *j* ≠ *i*, we have *e*_*i*,*j*_ = 1 if *i* = *i*_*α*_ or *j* = *i*_*α*_, and *e*_*i*,*j*_ = 0 otherwise.
5. For the high-level connections between subgroups, the global center image *I*_*o*_ is connected to all other representative images $I_{i_{\alpha }}$ that are determined in Step (3). For any pair of images $I_{i}\;and\;I_{j}\in \{I_{i_{\alpha }}|\alpha =1,\ldots ,\Omega \}$ and *j* ≠ *i*, the conclusion can be drawn that *e*_*i*,*j*_ = 1 if *i* = *o* or *j* = *o*, and *e*_*i*,*j*_ = 0 otherwise. An example graph is shown in Fig 1.

We can assume that all images within each subgroup are structurally similar. Thus, the image closest to the global center can be chosen as the representative image to guide deformations of all images in the subgroup to the global center image.

### Graph-Shrinking-Guided Image Normalization

We assure that all images are connected, and therefore the graph has a total of *N* − 1 edges. Based on this graph, all images in the manifold can be warped in accordance to their connected images. The registration process can then be viewed as a dynamic graph shrinkage process, as described in our previous HUGS (Hierarchical Unbiased Graph Shrinkage) for groupwise registration [23]. A brief description of HUGS is given here.

In general, we can assume the deformation of each individual image as a dynamic procedure of time variable *t*. Thus, *I*_*i*_(*t*) can be used to represent the deformed image *I*_*i*_ at time *t*. Next, we use a graph defined in the brain image manifold as follows. Let $I(t)={\left\{I_{i}(t)\right\}}_{i=1}^{N}$ be the graph nodes and *E* = {*e*_*ij*_: *i*,*j* = 1, …, *N*} be the edges between two nodes in the graph. *e*_*ij*_ = 1 means existence of a link between *I*_*i*_(*t*) and *I*_*j*_(*t*). Otherwise, there is no direct link between *I*_*i*_(*t*) and *I*_*j*_(*t*) in the graph. Also, we define a *N* × *N* weighted adjacency matrix where each element exp (*v*_*ij*_(*t*)) describes the geodesic pathway between two images, such that *v*_*ij*_(*t*) > 0 if *e*_*ij*_ = 1 and *v*_*ij*_(*t*) = ∞ otherwise. Note that *v*_*ij*_(*t*) denotes the velocity vector of geodesic pathway, which indicates the distance between *I*_*i*_(*t*) and *I*_*j*_(*t*). Here, deformable image registration [12] is performed to estimate each velocity vector *v*_*ij*_(*t*) and further calculate the deformation pathway between *I*_*i*_(*t*) and *I*_*j*_(*t*) by exp (*v*_*ij*_(*t*)), where ‘exp’ is the exponential map [33]. Intuitively, the goal of our graph-based groupwise registration is to minimize the velocity vectors along all graph edges, as defined below:

$$F(t)=\sum_{i,j=1}^{N}e_{ij}{\left\|v_{ij}(t)\right\|}^{2}.$$ (3)

**Fig 3:**
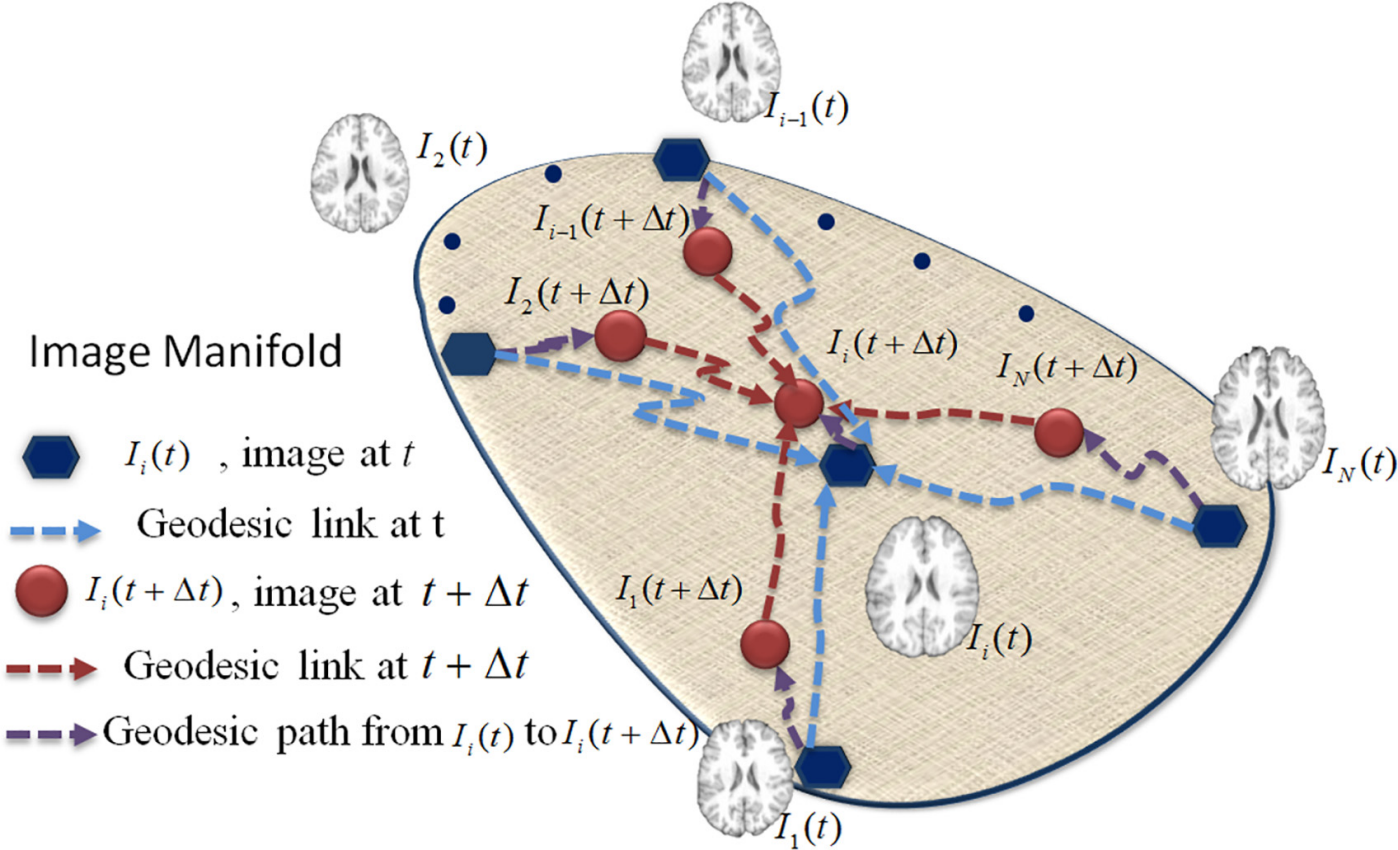
Registration via graph shrinkage. At time t, all the images in the graph (denoted by blue points) are connected to each other by the geodesic path (denoted by blue dash lines). At time t+Δt, all the images are deformed according to their connected images through geodesic paths (denoted by purple dash lines). The deformed images (denoted by red points) become closer to each other and a new graph describing their relationship is generated (denoted by red dash lines).

The principle behind *F*(*t*) is demonstrated in **Fig 3**. First, all images are assumed to be sitting in a high-dimensional manifold. Then, the topology of their distribution is described by a graph, where the graph edges denote the local connectivity between graph nodes. Specifically, the velocity vector *v*_*ij*_(*t*) is associated with each graph edge, where the integration along *v*_*ij*_(*t*) forms the geodesic distance from *I*_*i*_(*t*) to *I*_*j*_(*t*). Thus, the minimization of *F*(*t*) can be regarded as a dynamic graph shrinking procedure, which deforms each image from *I*_*i*_(*t*) to *I*_*i*_(*t* + Δ*t*) with the decreased overall geodesic distance, while keeping the topology of the entire graph. As time *t* increases, all *I*_*i*_(*t*)s are supposed to meet at the population center, with properly determined velocity vector *v*_*ij*_(*t*) and time increment Δ*t*.

As we formulate the problem of groupwise registration as the dynamic shrinkage of graph, it is critical to determine the deformation of each image *I*_*i*_(*t*) at time *t*, which can minimize energy function *F*(*t*). Suppose each image has been deformed from *I*_*i*_(*t*_0_) to *I*_*i*_(*t*_*k*_), where {*t*_*k*_} is the discretization of time *t* (*k* = 0, … *K*, *t*_0_ = 0, *t*_*K*_ → ∞). Regarding the local connectivity of each node *I*_*i*_(*t*_*k*_) in the graph, it is reasonable to move *I*_*i*_(*t*_*k*_) along the average direction according to its connected nodes. Since the velocity vector sits on the tangent space of *I*_*i*_(*t*_*k*_) on the manifold, it can be efficiently calculated by linear averaging as ${\hat{v}}_{i}(t_{k})=\frac{1}{N_{i}}\sum_{j=1}^{N}e_{ij}v_{ij}(t_{k})$, where $N_{i}=\sum_{j=1}^{N}e_{ij}$ is the number of connections for *I*_*i*_(*t*_*k*_). Given the direction of each velocity vector ${\hat{v}}_{i}(t_{k})$ at time point *t*_*k*_, it can be proven that the entire energy function *F*(*t*) is strictly and monotonously decreased if each node *I*_*i*_(*t*_*k*_) moves along the direction of velocity vector ${\hat{v}}_{i}(t_{k})$ from *t*_*k*_ to *t*_*k*_ + Δ*t*_*k*_, where the time increment Δ*t*_*k*_ is bounded by $\Delta t_{k}=min\left\{\frac{1}{{max}_{i}\|{\hat{v}}_{i}(t_{k})\|},\frac{\sum_{i=1}^{N}N_{i}{\left\|{\hat{v}}_{i}(t_{k})\right\|}^{2}}{\sum_{i=1}^{N}\left(N_{i}+1\right){\left\|{\hat{v}}_{i}(t_{k})\right\|}^{2}}\right\}$. Eventually, the geodesic pathway *φ*_*i*_ from each image *I*_*i*_ to the population center can be obtained by concatenating the deformation segments from *t*_0_ to *t*_*K*_, i.e., $\varphi_{i}=exp({\hat{v}}_{i}(t_{K})∙∆t_{K})\circ \ldots \circ exp({\hat{v}}_{i}(t_{0})∙∆t_{0})$, where ‘∘’ denotes the deformation composition [34]. During graph shrinkage, all images are gradually deformed to the hidden common space with preservation of graph topology. For numerical implementation, we discretize *t* using *t*_*k*_ (*k* = 0,1,2,…, with *t*_0_ = 0 and *t*_*k*_ → ∞ as *k* → ∞) based on the interval Δ*t*. Then we use $I_{i}^{k}$ as the new deformed image at time *t*_*k*_, and also ${\hat{v}}_{i}^{k}$ as the composited deformation field of image $I_{i}^{k}$ at time *t*_*k*_.

### Summary of Our Method

Our method is briefly summarized as follows:

1. Perform affine registration to obtain a set of *N* affine-aligned images ***I*** = {*I*_*i*_|*i* = 1, …, *N*}.
2. Compute all distances *d*_*i*,*j*_ = ‖*I*_*i*_ − *I*_*j*_‖^2^, ∀*i*,*j* ∈ {1, …, *N*}.
3. Cluster images ***I*** into *Ω* subgroups *G*_*α*_ ($\alpha \in \{1,\ldots ,\Omega \},\;and\;{⋃}_{\alpha =1}^{\Omega }G_{\alpha }=I$) by using affinity propagation.
4. Construct a null connection matrix *E* with elements *e*_*i*,*j*_ = 0, ∀*i*,*j* ∈ {1, …, *N*}.
5. Obtain the global center image *I*_*o*_ by Eq 2.
6. Determine in each subgroup *G*_*α*_ the image nearest to the global center image *I*_*o*_ as the representative image $I_{i_{\alpha }}$.
7. Establish intra-subgroup connections in the same subgroup by connecting all images *I*_*i*_ {*I*_*i*_ ∈ *G*_*α*_ and *i* ≠ *i*_*α*_} with the representative image $I_{i_{\alpha }}$. Then, establish inter-subgroup connections by connecting all representative images $\left\{I_{i_{\alpha }}|\alpha =1,\ldots ,\Omega \;and\;i_{\alpha }\neq o\right\}$ and the global center image *I*_*o*_.
8. Perform registration via graph shrinkage iteratively until convergence.

## Results and Discussion

eHUGS offers an efficient and accurate solution to tackle heterogeneous image datasets (We found eHUGS did not achieve significant improvement over HUGS on small dataset such as IXI (30 subjects) and NIREP (16 subjects) since single graph is sufficient to describe the image distribution for small number of subjects). We evaluate the proposed method on both adult and infant datasets. Specifically, we tune the parameters of our eHUGS registration method using the 4-time-point longitudinal brain MR scans of 20 elderly normal subjects, obtained from the ADNI dataset [27]. Then, using these tuned parameters, we apply eHUGS to the infant dataset, which includes 285 MR brain images of infant subjects with 2 weeks to 2 years of age. The proposed method is compared with the standard group-mean method [25], ABSORB [17], and HUGS [35].

### Experiments on Elderly Brains

**Fig 4:**
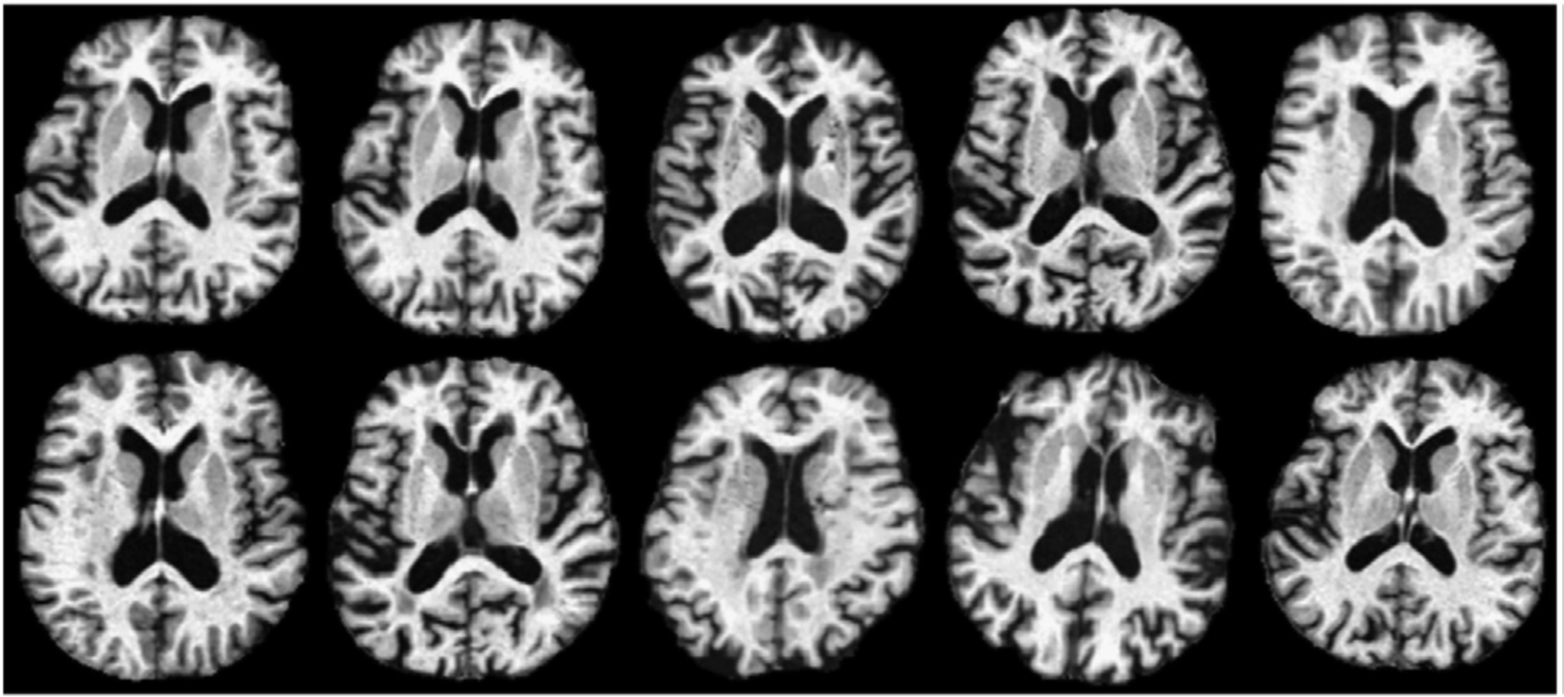
Image samples of elderly subjects obtained from the ADNI database. In this figure, large variations across subjects can be observed.

In this experiment, 20 normal subjects are chosen from the ADNI database. In addition to the baseline scan, each subject was also scanned after 6 months, 1 year, and 2 years. Sample baseline scans are shown in Fig 4. This dataset is preprocessed using the following steps. First, anterior commissure (AC) posterior commissure (PC) correction is applied to all images, which are further resampled to size 256×256×256 with voxel resolution 1mm×1mm×1mm. After applying the N3 algorithm [36] for intensity inhomogeneity correction, Brain Surface Extractor (BSE) [37] and Brain Extraction Tool (BET) [38] are used for skull stripping. Next, each image is segmented into three types of tissues: white matter (WM), gray matter (GM), and cerebrospinal fluid (CSF) by using FAST [39] in FSL software package (http://fsl.fmrib.ox.ac.uk/fsl/fslwiki/). We further visually inspect and manually correct the segmentation results. Hence, we can regard these segmentation results as the ground truth in evaluating the registration accuracy. Finally, affine registration using FLIRT [28] is applied.

**Fig 5:**
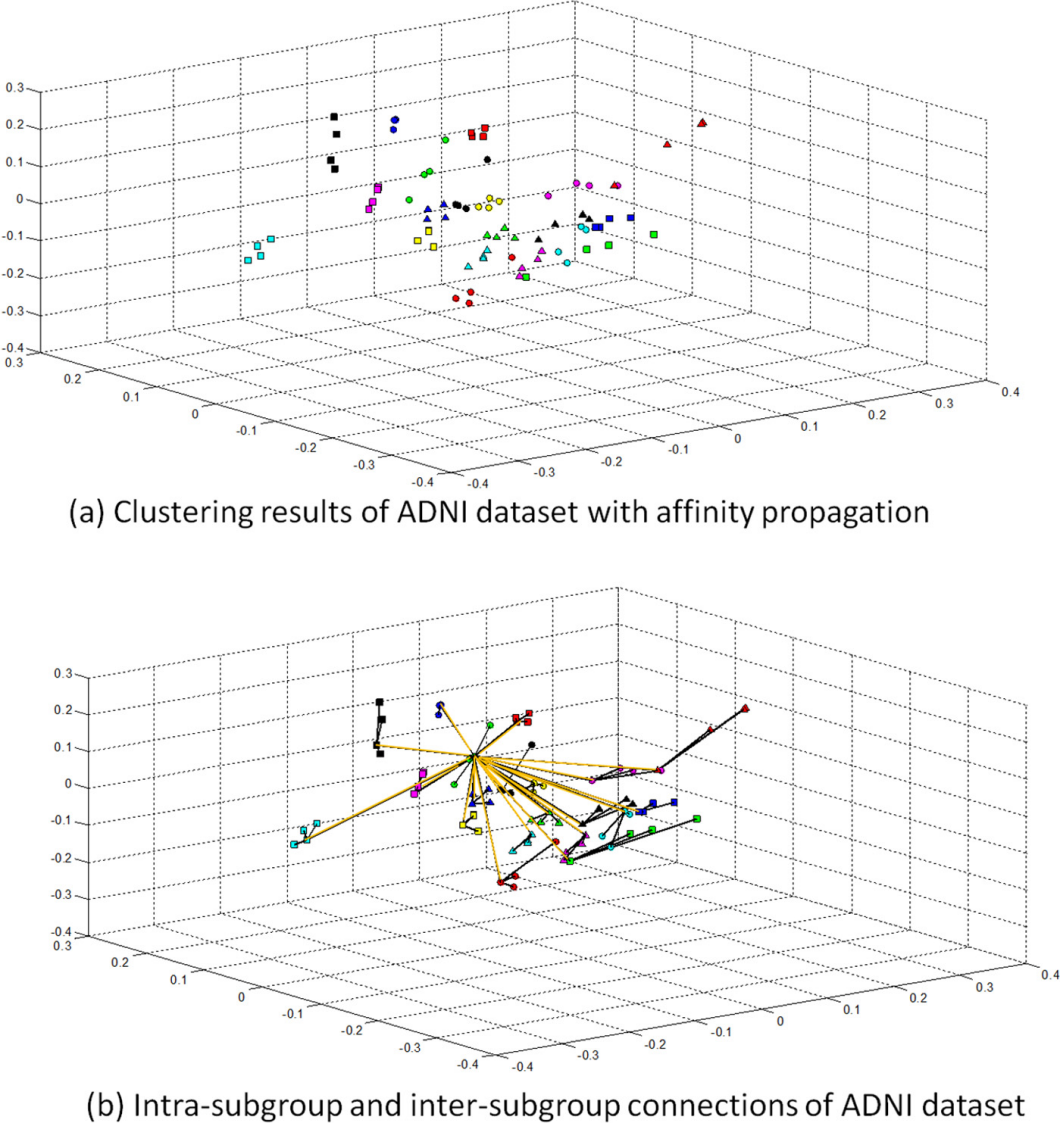
Clustering and graph construction. (a) Images are clustered into 20 subgroups, represented with different markers and colors, by using affinity propagation. (b) Intra-subgroup (black color) and inter-subgroup (orange color) connections in the graph.

In eHUGS, the distance matrix is calculated using the pre-processed brain images and then affinity propagation is applied to cluster all the images. In this way, we obtain 20 subgroups based on the observed image dataset. The clustering result is shown in Fig 5 (A), where all images have been projected to a 3-dimensional space. Each dimension of the projected space denotes one of the 3 largest eigenvectors obtained by performing Principal Component Analysis (PCA) on the image distribution. Both intra- and inter-subgroup connections are also shown in Fig 5 (B).

Evaluations of eHUGS, HUGS, ABSORB, and the standard group-mean method are performed on a Dell workstation with 2 Xeon E74850, 2.0Ghz, 10 core CPU, and 256GB/1066Mhz RAM. The computation times of all methods are summarized in Table 1. There are in total 79 edges in the graph constructed in eHUGS, compared with 1265 edges in the graph given by HUGS. This causes HUGS to be nearly 20 times slower than eHUGS. The standard group-mean method gives 79 edges, and ABSORB gives more than 79 edges. Generally, in terms of computation time, eHUGS ≅ group-mean < ABSORB < 1-week computation time< HUGS (~1 month).

**Table 1:** Computation times of the standard group-mean method, ABSORB, HUGS, and eHUGS on the elderly brain image dataset, obtained from ADNI.

	Time (hour)
**Group-mean**	40
**ABSORB**	72
**HUGS**	640
**eHUGS**	48

To quantitatively measure registration accuracy, the Dice ratio is used to measure the degree of overlap of a specific type of tissue between different images after registration. The Dice ratio is defined as

$$\text{Dice}\left(A,B\right)=2\times \frac{\left|A\cap B\right|}{\left|A\right|+\left|B\right|}.$$ (4)

Here, | ∙ | is the voxel count within the region-of-interest. *A* and *B* denote the regions spanned by a specific type of tissue, respectively, in the two images. Better registration should yield higher Dice ratios [40]. Since no explicit template image is selected in groupwise registration, the ground-truth segmentation in the common space is obtained by majority voting from all aligned images. Then, the Dice ratio associated with each tissue type (WM, GM, and CSF) is computed with respect to the estimated ground-truth segmentation.

**Fig 6:**
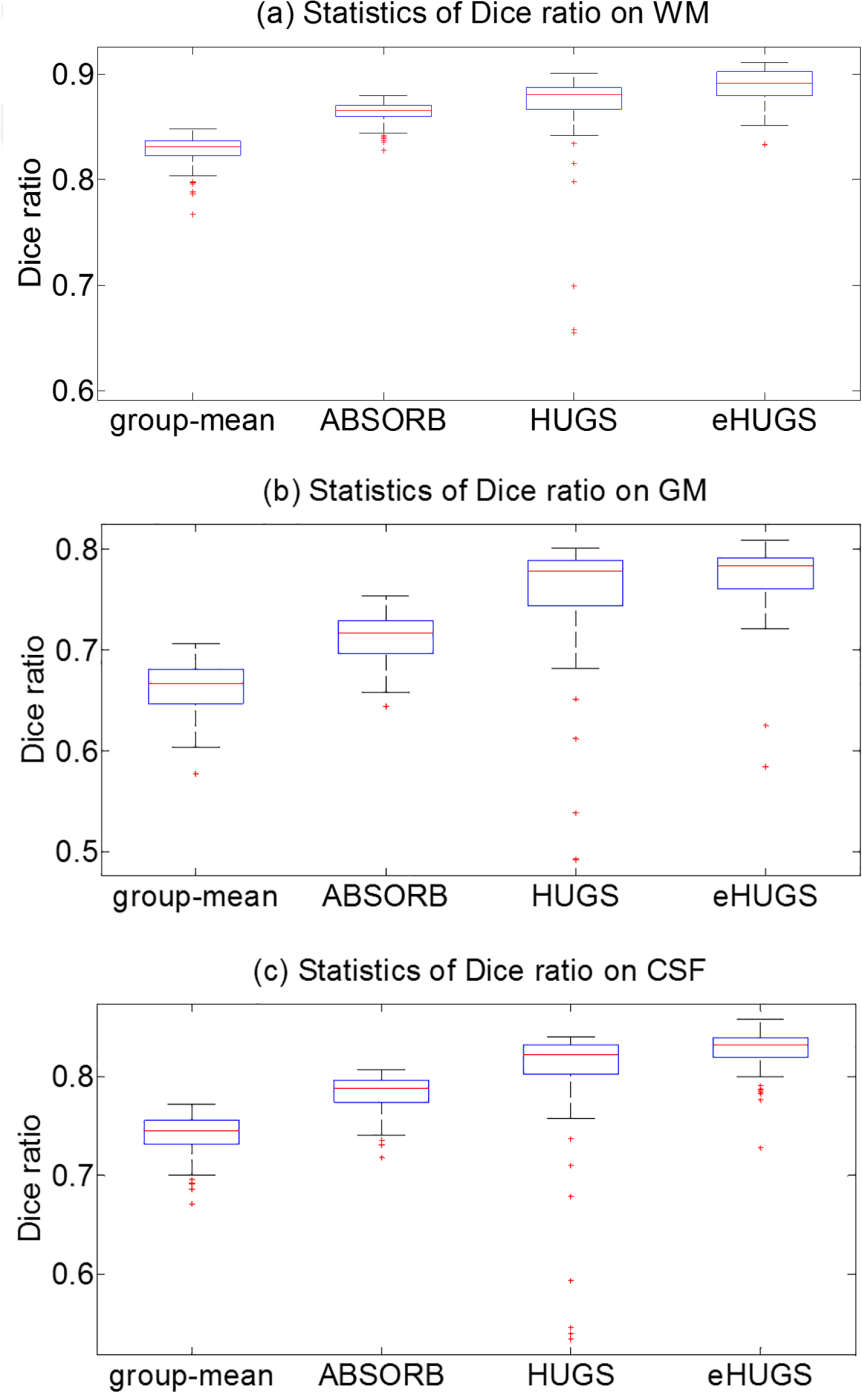
The box plots of Dice ratios of WM, GM and CSF for the elderly brain dataset (ADNI). (a) Dice ratio produced by the standard group-mean method. (b) Dice ratio produced by ABSORB. (c) Dice ratio produced by HUGS. And (d) Dice ratio produced by eHUGS.

Before (non-rigid) groupwise registration, the average Dice ratios of WM, GM, CSF, and all tissues in overall (after affine registration) are 70.01±2.99%, 55.20±3.00%, 60.64±2.81%, and 61.95±2.93%, respectively. Table 2 shows the average Dice ratios of each tissue type given by the standard group-mean method, ABSORB, HUGS, and eHUGS, respectively. The lower quartiles, medians, and upper quartiles of Dice ratios on WM, GM and CSF are also shown in Fig 6(A)–6(C), respectively, for different registration approaches. It can be observed that our eHUGS achieves the highest Dice ratio for each tissue type. Since the conventional group-mean registration method independently registers each individual image to the population center, such outlier images undermine the group mean and also mislead the registration result of whole group. On the contrary, our graph-based groupwise registration method adaptively deforms each image based on the neighbouring images in the high-dimensional image manifold, with the goal to improve the overall registration accuracy while minimize the influence from the outlier images.

**Table 2:** Dice ratios of WM, GM, CSF and all tissues, obtained by the standard group-mean method, ABSORB, HUGS, and eHUGS, respectively, for the elderly brain dataset (ADNI).

	WM	GM	CSF	Overall
**Before Non-linear Registration**	70.01±2.99%	55.20±3.00%	60.64±2.81%	61.95±2.93%
**Group-mean**	82.62±1.64%*	66.18±2.77%*	74.07±2.14%*	74.29±2.18%
**ABSORB**	86.32±1.15%*	71.18±2.48%*	78.32±1.86%*	78.61±1.83%
**HUGS**	86.57±4.91%*	75.27±6.80%*	80.20±6.37%*	80.68±6.03%
**eHUGS**	88.58±3.53%	77.23±3.44%	82.64±2.04%	82.82±3.01%

**Fig 7:**
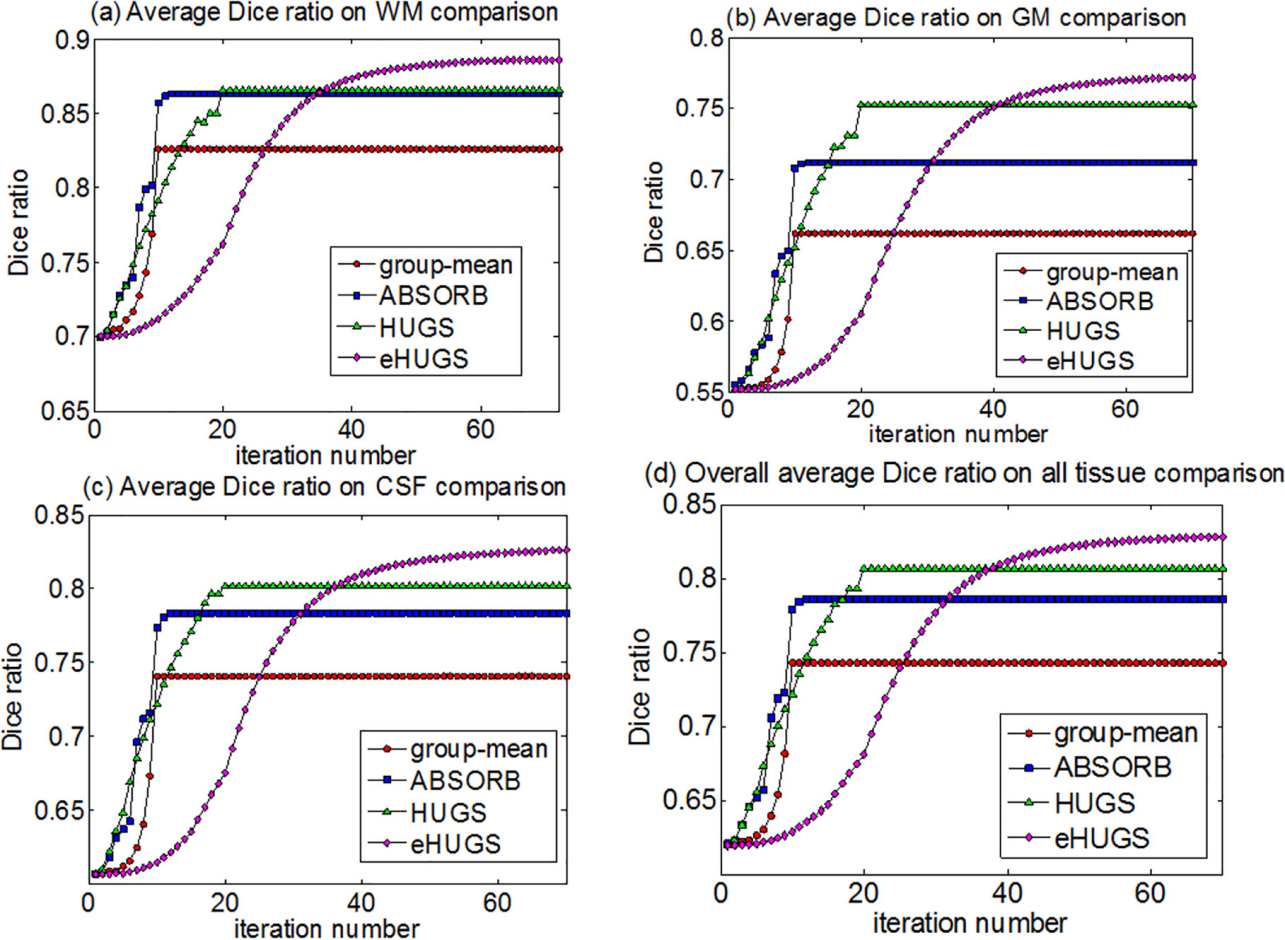
Changes of Dice ratios with progress of groupwise registration for the elderly brain dataset (ADNI).

The average Dice ratios of WM, GM and CSF obtained by the proposed method (eHUGS), HUGS, ABSORB, and the standard group-mean method in each iteration are shown in Fig 7(A)–7(D), respectively. For each method, the Dice ratio becomes stable after a certain number of iterations. From Fig 7(A)–7(D), we can observe that the average Dice ratios for WM, GM, CSF and all tissues in overall given by eHUGS are higher than any other method. We apply the paired *t*-test between our eHUGS and all other three counterpart methods, and our eHUGS achieves significant improvement over all three methods in terms of Dice ratio with *p* < 0.01. More strictly, we further apply Mann-Whitney U-test, after the false discovery rate (FDR) correction, and also find that the improvements by eHUGS over all other three groupwise registration methods are still statistically significant (*p* < 0.01) in all tissue types, as indicated by the red ‘*’ in Table 2.

It is worth noting that our graph-based groupwise registration is very flexible to work with other similarity measurements. Here, we also use mutual information (MI) to compute the pairwise similarity and construct the hierarchical graph. The Dice ratios in WM, GM, and CSF after groupwise registration using MI are 88.54%, 77.24%, and 82.49%, respectively, which are comparable to the results using SSD (last row in Table 2).

### Experiments on 285 Infant Brains (Aged from Birth to 2-year-old)

**Fig 8:**
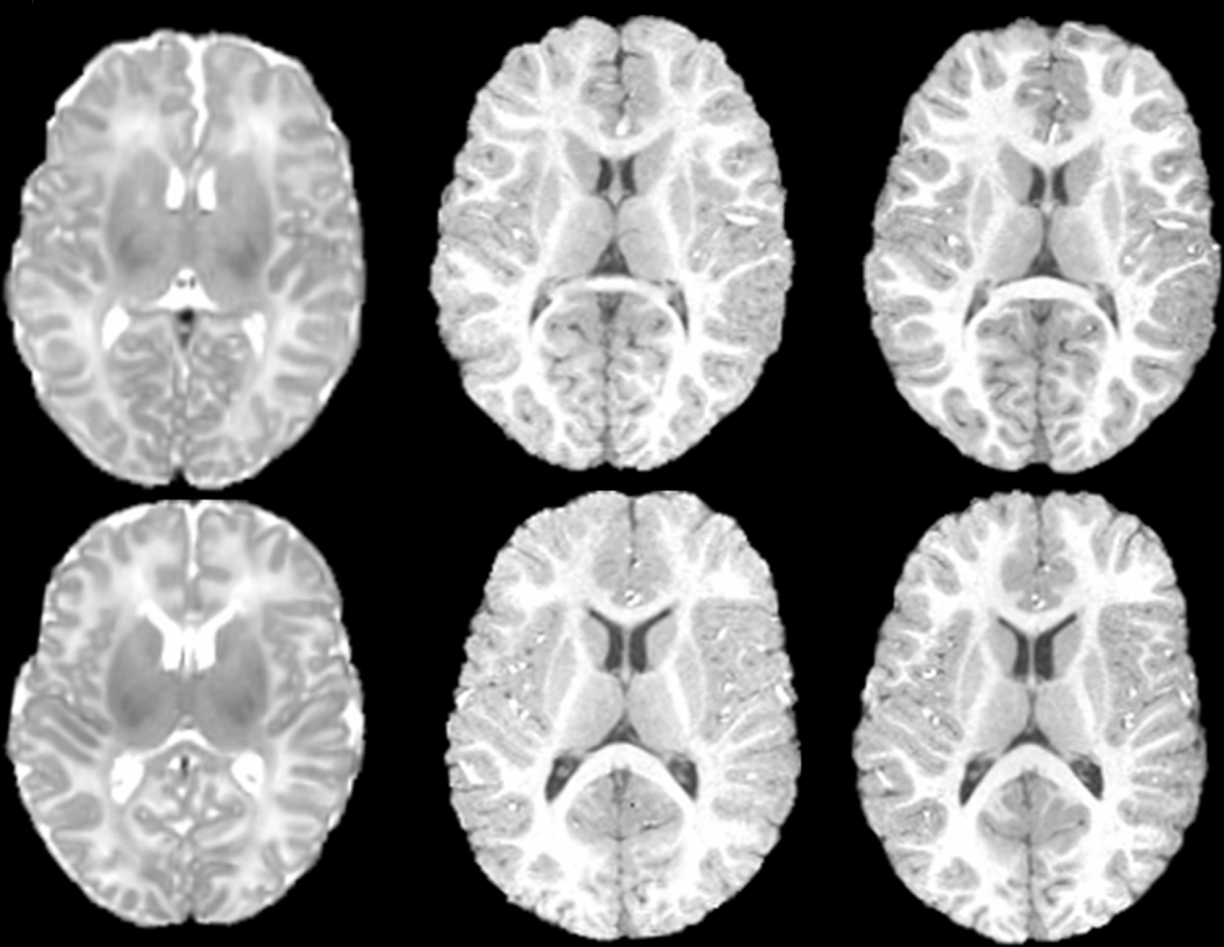
Sample images of two subjects from the infant database. We can observe large structural variation across subjects, and also large appearance variation across different images of same subject.

In this experiment, we evaluate the groupwise registration result on 95 longitudinal infant image series. Each infant subject has been scanned at birth, 1 year old, and 2 years old. All these images were acquired using a Siemens head-only 3T scanner for 95 subjects. Due to WM myelination, we use T2-weighted images for neonates and T1-weighted images for 1–2 year(s) old infants. For neonatal subjects (scanned at birth), we use T2-weighted images with the following parameters: TR = 7380ms, TE = 119ms, flip angle = 150°, acquisition matrix = 256×128, and voxel resolution = 1.25×1.25×1.95mm^3^. For 1-year-old and 2-year-old subjects, we use T1-weighted images, with the following imaging parameters: TR = 1900ms, TE = 4.38ms, flip angle = 7°, acquisition matrix = 256×192, and voxel resolution = 1×1×1mm^3^. T2-weighted images are affine aligned onto the respective 1-year-old T1-weighted images of the same subject and further resampled to 1×1×1mm^3^. All images are cropped to the size 256×256×198. Then skull stripping, inhomogeneity correction are performed to all T1- and T2-weighted MR images. Since we have each infant subjects scanned in 0-year-old, 1-year-old, and 2-year old, we employ the state-of-the-art infant segmentation method [41] to obtain reasonable segmentation results for infant images by letting the segmentation of later time points guide the challenging segmentation in the 0 year old. We further manually inspect and correct the segmentation result, in order to guarantee the segmentation quality in evaluating the registration accuracy. After that, we perform FLIRT in FSL package to linearly register all infant subjects to the common space. As shown in Fig 8, structural variations across different infants are large, and also the appearances across different times of same infant are also large.

**Fig 9:**
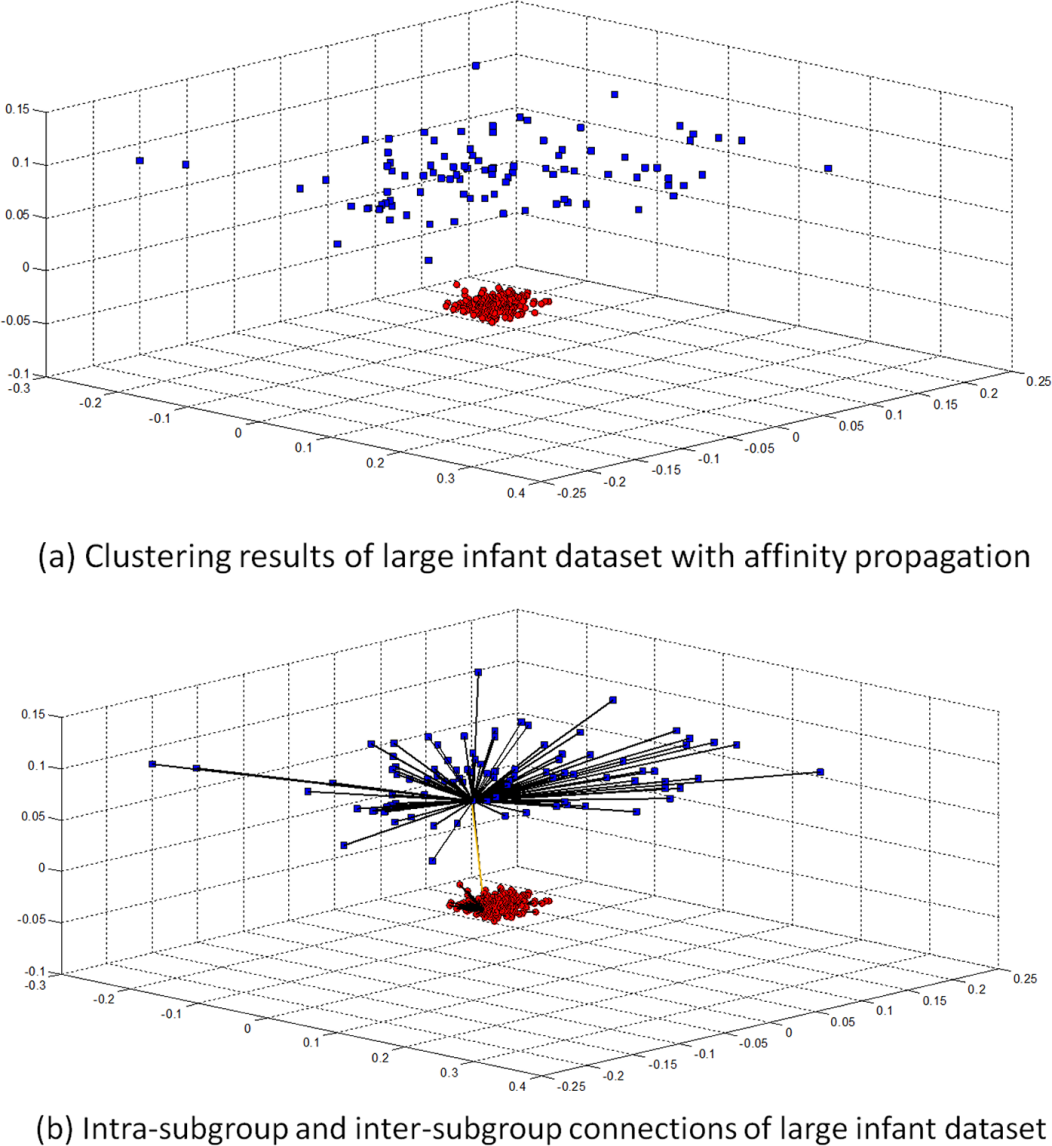
Clustering and graph construction. (a) Images are clustered into 2 subgroups, represented with different markers and colors, by using affinity propagation. (b) Intra-subgroup (black color) and inter-subgroup (orange color) connections in the graph.

In eHUGS, again the distance matrix is calculated for the pre-processed brain images, and then affinity propagation is applied to cluster the images, resulting in 2 subgroups. As can be seen from Fig 8, the images of 1-year-olds and 2-year-olds are relatively similar, especially when compared with their differences with those of neonates. This is consistent with the fact that the total brain volume increases 101% in the first year of life, in contrast to only 15% in the second year (Knickmeyer, et al., 2008). The clustering result is shown in Fig 9 (A), where all images are projected to a 3-dimensional space. Again, each dimension denotes one of the 3 largest eigenvectors by performing PCA on the image distributions. The intra- and inter-subgroup connections are also shown in Fig 9 (B).

There are in total 284 edges in the graph constructed by eHUGS, compared with 24,494 edges in the graph given by HUGS. This causes HUGS to be nearly 84 times slower than eHUGS. In fact, for this dataset, based on our estimation, HUGS needs more than a year to complete the registration. In this case, we only compare eHUGS with the standard group-mean method and ABSORB. For all methods, the computation times are summarized in Table 3.

**Table 3:** Computation times of the standard group-mean method, ABSORB, HUGS, and eHUGS for the infant brain image dataset.

	Time (hour)
**Group-mean**	170
**ABSORB**	236
**HUGS**	185×84 (estimate)
**eHUGS**	185

**Fig 10:**
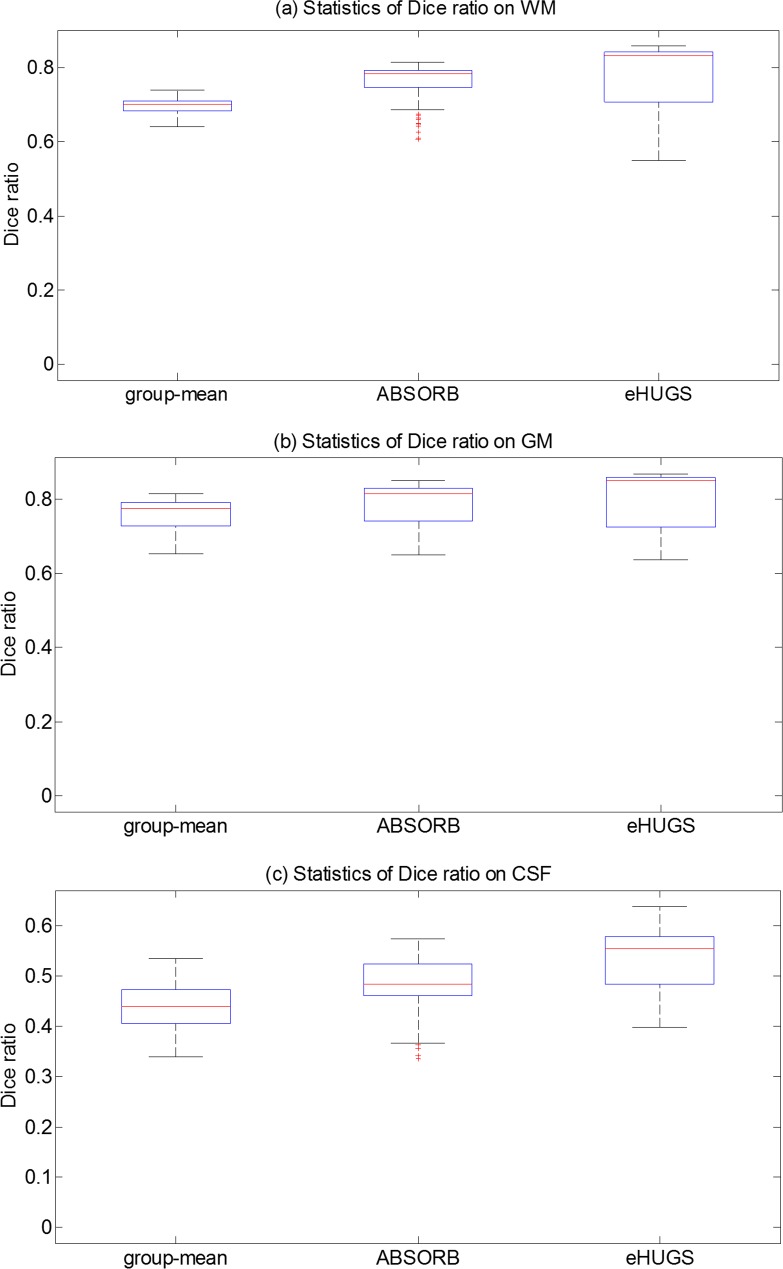
The box plots of the Dice ratios of WM, GM and CSF for the infant dataset. (a) By the standard group-mean method. (b) By ABSORB. (c) By eHUGS.

Before (non-rigid) groupwise registration, the average Dice ratios of WM, GM, CSF, and all tissues in overall are 60.93±9.15%, 72.29±11.11%, 39.19±7.57%, and 57.47±9.28%, respectively. Table 4 shows the average Dice ratio of each tissue type. The lower quartiles, medians, and upper quartiles of Dice ratios on WM, GM and CSF are shown in Fig 10(A)–10(C), respectively, for different registration approaches. It can be observed that eHUGS again achieves the highest Dice ratio in all comparisons.

**Table 4:** Dice ratios of WM, GM, CSF and all tissues, obtained by the standard group-mean method, ABSORB, and eHUGS, respectively, for the infant dataset.

	WM	GM	CSF	Overall
**Before Non-linear Registration**	60.93±9.15%	72.29±11.11%	39.19±7.57%	57.47±9.28%
**Group-mean**	68.18±10.24%	74.53±11.57%	43.16±7.61%*	61.96±9.81%
**ABSORB**	76.69±14.97%	78.79±12.78%	48.47±10.44%*	67.98±12.73%
**eHUGS**	77.44±13.12%	78.88±13.56%	52.79±9.59%	69.70±12.09%

**Fig 11:**
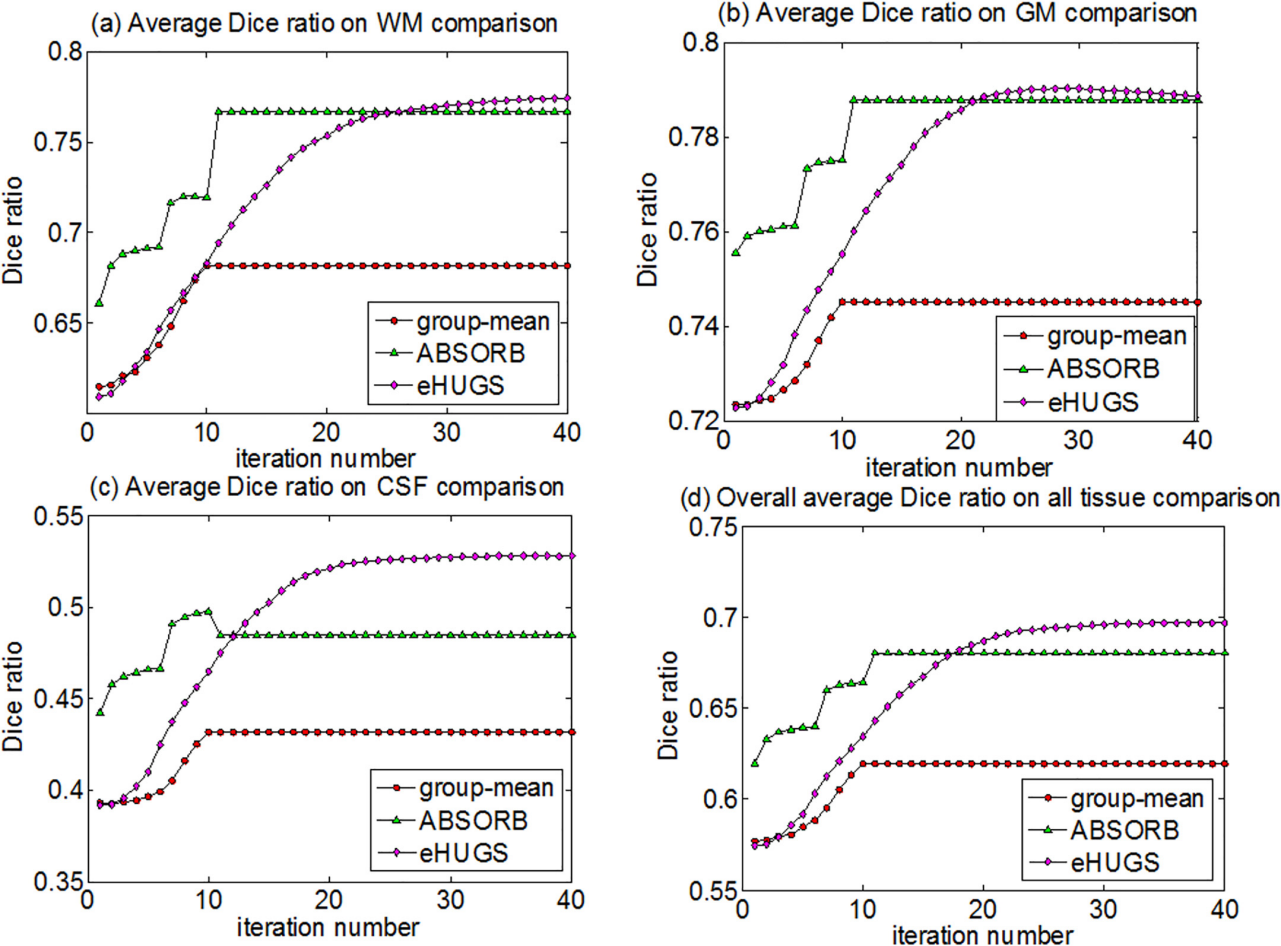
Changes of Dice ratios with progress of groupwise registration for the infant brain dataset.

The average Dice ratios of WM, GM and CSF obtained by the proposed method (eHUGS), the standard group-mean method, and ABSORB in each iteration are shown in Fig 11(A)–11(D). From Fig 11(A)–11(D), we can draw similar conclusions as for the elderly brain dataset. That is, the average Dice ratios on WM, GM, CSF and the overall by the proposed method are higher than any other method. Under paired *t*-test, we find that our eHUGS achieves significant improvements (*p* < 0.01) over group-mean, and ABSORB in terms of Dice ratio on all tissue types. By considering all 285 aligned images together, we also perform the Mann-Whitney U test between the Dice ratios of eHUGS and all other methods. After FDR correction, the multiple comparison tests show that eHUGS achieves significant improvement in CSF with *p* < 0.01, as indicated by the red ‘*’ in Table 4. Similarly, we also replace the SSD measurement with MI in constructing the graph. The final groupwise registration results by using two image similarity measurements are comparable, where the tissue overlap ratios in WM, GM, and CSF are (77.44%, 78.88%, 52.79%) by using SSD and (77.96%, 78.64%, 52.96%) by using MI.

**Fig 12:**
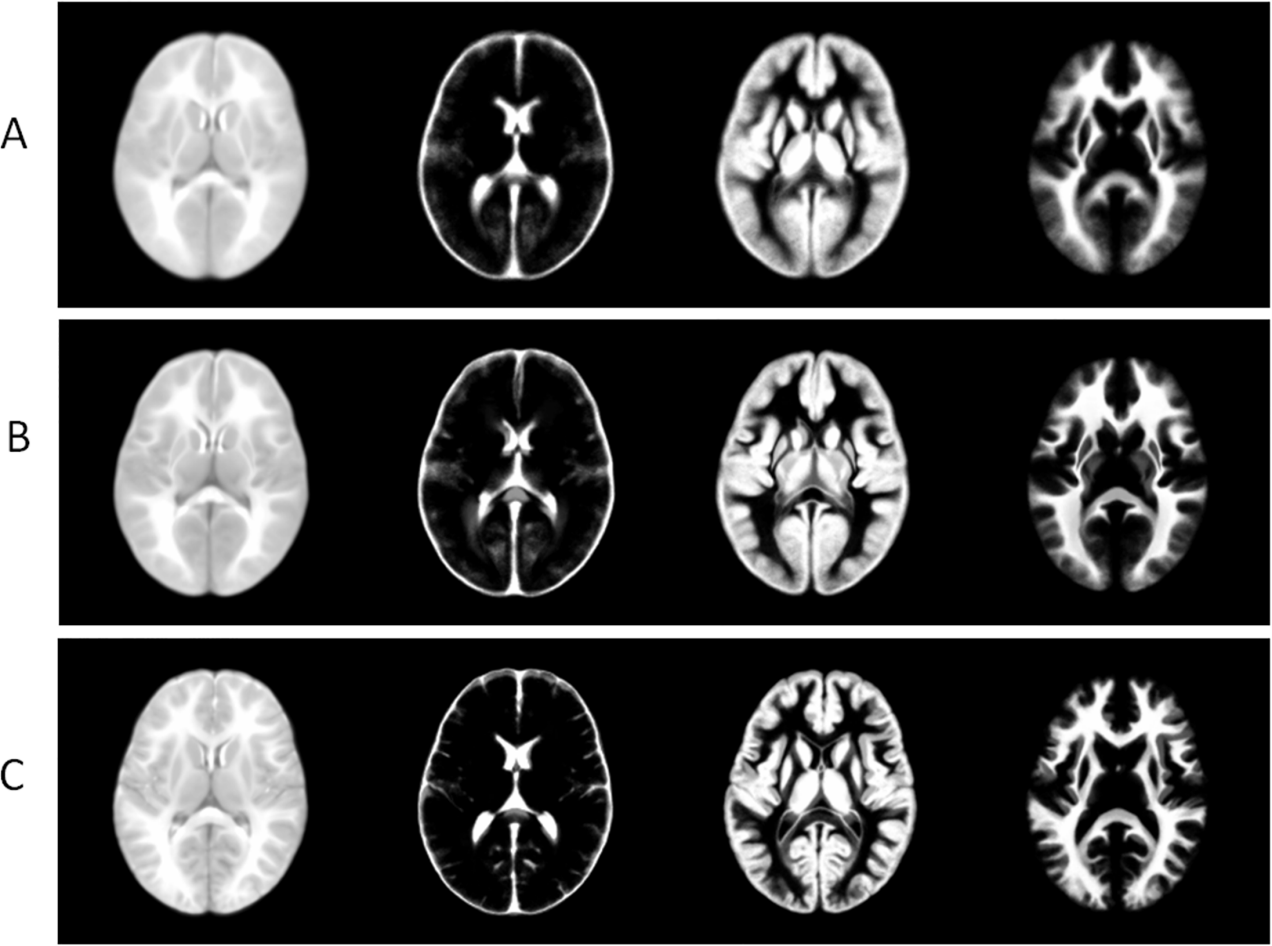
Illustration of the atlases of infant brains. A-C are obtained by the standard group-mean method, ABSORB, and eHUGS, respectively. In each panel, from left to right are the mean image, and tissue probability maps for CSF, GM and WM, respectively.

Since the infant atlas is very important in many brain development studies, we specifically evaluate the registration accuracy by inspecting the quality of the infant atlases, constructed from all aligned infant images. Here, we use a simple averaging to get the mean image, and use majority voting to generate the tissue probability maps for WM, GM, and CSF, respectively. From top to bottom, in Fig 12, we show the constructed atlas (mean image, WM, GM, and CSF probability maps) by the standard group-mean method, ABSOB, and eHUGS. Through visual inspection, the atlases constructed by eHUGS show greater anatomical details than any other method.

## Conclusion

In this paper, we have presented an effective and efficient method for unbiased groupwise registration of large dataset of images. Specifically, we propose an enhanced version of HUGS, namely eHUGS, to model the complex image distribution by using the hierarchical graph and thus overcome the limitation of using a single graph in HUGS. Experiments using both elderly and infant brain images indicate that eHUGS gives the improved registration performance. In all experiments, eHUGS yields the best Dice ratio when compared with the standard group-mean method, ABSORB, and HUGS.

In our proposed eHUGS, our main idea is to use hierarchical graph for representing image distribution, and then register images progressively to each other for avoiding direct registration between different images. For our current datasets, using simple image similarity metric, we found that 2 levels are enough to represent the image distribution. But, for other datasets, more levels may be needed for representing more complex image distribution. On the other hand, when affine aligning all images to the comment space, although we select a population center as a template, pairwise registration is still used. Potentially, we can use groupwise affine registration for alignment of the whole dataset, which could improve the registration results of all affine-aligned images. All these will be our future work.

Besides, our other future work also includes (1) evaluating the registration performance of eHUGS on large datasets; (2) applying eHUGS to other applications, such as group comparison for discovering AD imaging biomarkers.
